# On Medical Reform

**Published:** 1810-01

**Authors:** 


### ON MEDICAL REFORM.

I HAVE been an attentive, and not an unconcerned Observer, of what had been written and attempted on Medical Reform, long before the question was first agitated by the Lincolnshire Benevolent Medical Society. Not that I should have now taken up my pen, had I not perceived that the object of the Reformers, and proceedings adopted, have been completely mistaken in several instances by one Writer, in the preceding Number of the Journal, who calls himself quaintly, and to me I confess unintelligibly, "An Enemy to the Quackery of Corporate Bodies."

In Dr. Harrison's last circular Letter, he remarks, that "Quacks, aware of their ignorance and misdeeds, artfully conceal them, by compounding and dipensing their own drugs." Now, because Apothecaries happen to dispense medicine, this Writer infers, that an useful and highly meritorious class of Practitioners are intended to be confounded, and of course stigmatised, in common with empirical Pretenders. Had he taken the trouble to read the whole Letter, and compare it with the other circular Addresses, he would have seen that nothing of the kind was ever meant. The regular establishment, viz. Physicians, Surgeons, Apothecaries, and Men-Midwives, are every-where carefully distinguished from Quacks, or uneducated Pretenders. I may further observe, that during Dr. Harrison's late visit to London, I had an opportunity of examining the Bill; and I can venture to assure the Profession, that no *practical* restraints are laid upon Apothecaries, or other Members of the Faculty. The Bill requires a suitable education for the different orders of the Profession, but does not attempt to limit their exertions. In small towns, and among the poor, Apothecaries must continue to undertake every branch of the healing-art; or the sick will, in many cases, be wholly deprived of medical aid. Any reform, which professes to confine the practice of the different orders of the Profession by legal enactments, would, in my opinion, be highly injurious to the community. I am happy, therefore, in being able to assert, of my own knowledge, that nothing of the kind is attempted in the Bill; and I think the ingenious and learned Editors of the Medical and Physical Journal can confirm my statement; since, unless I am misinformed, the Bill has been submitted to their consideration, in common mon mon with many others. The same Writer does not hesitate to assert, that the attainments of the Regular Faculty are much greater, and the number of empirical pretenders much less, than formerly. I am ready to admit that Medical Men, of great learning and superior professional endowments, are to be met with in all parts of the empire; but I am far from believing that the great body of the Faculty are as well instructed in classical or medical learning, as their immediate predecessors. Nor do I think that their emoluments enable them to maintain their former style of living, or that they are now placed by others in their proper rank. The remark of Viator is, I believe, strictly true; that Physicians are now placed on the list of precedence below Naval Lieutenants. Formerly Doctors, in all the Faculties, held a distinguished and an equal situation. In Divinity and Law, the old rank is carefully preserved. Why has a change, so degrading to the medical character, been suffered by the Fraternity; for as one class sinks, the others must proportionally descend? It is owing, as I conceive, to the disorganised state of the Profession; which, by suffering half-educated and illiterate Practisers to exercise the healing-art, has forfeited the pretensions of Medical Men to the first rank in human estimation.

Not a century ago Physicians, and Surgeon-Apothecaries, stood on an equality with Barristers and Attornies. But while Lawyers, in consequence of judicious regulations, have elevated their Members into Prime-Ministers, Chancellors of Universities, Senators, and Personages, of importance every-where—Medical Men, for want of union, have sunk in an equal degree. I have lived long enough in the world to mix with two generations; and such is the opinion I have formed from personal observation. With respect to Quacks, he asserts, that few of them are to be found out of London. Where this Gentleman has gained his information, is unknown to me. We are certainly completely at variance on this point. It has been my lot to visit many parts of the empire; and I have been led to believe, that this mischievous class is not only very abundant in country towns and villages, but that they are increasing in numbers and audacity. It has been stated on calculation, by the Lincolnshire Benevolent Medical Society, that Quacks exceed the regular Faculty, in the proportion of nine to one *. Whether the same inequality obtains

* See Dr. Harrison's Essay on the Ineffective State of Medical Practice.

obtains in other counties, may be doubtful. The question for consideration is simply, Whether it be for the interest of mankind, that Quacks should be tolerated or suppressed? And, 2dly, Whether the regular establishment ought to be subjected to rule, or left, as formerly, to the caprice of individuals.

The British is, I believe, the only Government in Europe, where Medical Men are suffered to enter upon their functions, without being previously licensed. It may be answered, that since the British are greatly superior to the continental Faculty, it will be to the interest of society to leave them to themselves. Admitting this boasted superiority, which is, however, very questionable, does it follow that they would not become more excellent, by obliging them to be suitably educated and examined.

The advantages obtained in the other learned Faculties, by regulations lately imposed, are to me a convincing proof, that, by similar means, the curative art would be greatly improved to the Profession and their employers. Under this impression, I am desirous to see the Bill carried into effect; and hope, before the winter business commences, that the pecuniary subscriptions will be sufficiently liberal, to meet the heavy expenses in Parliament.

Dr. Harrison is, I perceive, called upon by the same Writer, to publish the whole Bill for general information. This Gentleman is not perhaps aware, that such proceeding is unparliamentary, and would not be approved in that August Assembly.

After the second reading in the House of Commons, all Bills are ordered to be printed for distribution; and, at a future period, are taken into consideration by a Committee. In this interval, the Faculty will have time to examine the different parts, and to propose alterations by means of their representatives. I trust a copy of the Bill will be published in the Medical and Physical Journal, for general information.

The charge of Sir James Mansfield, and the legal opinion of Mr. Sergeant Williams, are most important documents, deserving the particular attention of Medical Corporations, and of all Country Practisers.

We are informed by the Chief Justice, contrary to a received opinion, that Diplomas, in themselves, confer no title to exercise Medicine; and, by the learned Serjeant, that the London College of Physicians possess no authority to controul Medical Practice, beyond seven miles from London.

In this dilemma, what is to be done? It is abundantly evident, that the great body of Doctors will never voluntarily apply for licenses to the College of Physicians; and it is equally clear, that so long as some Universities continue to sell their honors for money, regardless of other considerations, a large majority of provincial Physicians will be unequal to their important duties.

The obvious inference to be drawn from the above premises, as it strikes my mind, is, that a legal enactment is much wanted, to secure Invalids from the most dangerous Pretenders; from men, who, under the sanction of a purchased Diploma, can usurp the first rank and greatest responsibility in the Profession, however ignorant and undeserving. The College of Surgeons being founded on Charter, can only examine such as come voluntarily before them; hence few country Practisers think of applying to them for admission.

No Country Apothecaries enter into the Apothecaries' Company; consequently, a very numerous and highly meritorious part of the Profession, engage in all its branches, without exhibiting any proof of competence. No thinking person can approve of this laxity, which, by placing the able and the ignorant upon an equality, weakens the stimulus to meritorious exertion.

Of all descriptions in the commonwealth, Apothecaries give up the most time gratuitously; and are most unseasonably disturbed by their inconsiderate and selfish employers. When the Act is obtained, they will, of course, charge something for attendance and for consultations, in addition to the drugs had and used; by which means they will materially increase their emoluments, and be less intruded upon by the Public.

As soon as the influx of empirical, and other incompetent Persons, is prevented, by suitable restraints, the benefits will be immediately and strongly felt by all the Disciples of Æsculapius; and by none more than the Surgeon-Apothecary. He will daily rise in respectability. From being an inmate of the steward's-room, or the squire's humble companion, in the absence of other guests, he may look forward to become the familiar associate of his most fashionable patients.

Before concluding, it will be proper to notice the intended provisions in the Bill concerning Graduates; because it was observed by a Correspondent, in the last Journal, nal, that all persons possessed of a Diploma will be at liberty, according to the provisions of the new Bill, to practise as Physicians; but he forgot that the Diploma is, in future, to be coupled "in all cases with strict examinations;" and what is still more important, "the names, with the titles of the Faculty, and the University where the Diploma was obtained, are to be annually published in a register."

Under these several checks, I think few would venture hereafter to practise with an inferior Diploma; nor will any Medical Candidate be suffered to present himself before the examining boards, until he has passed through a suitable course of professional study.

It is by these mild and simple means that a Reform, useful to the people and honorable to the Faculty, is proposed to be established; and to its speedy accomplishment I sincerely say, Amen.

*Dec.* 14, 1809.

